# Supplementary material for: Resilience to abrupt global catastrophic risks disrupting trade: Combining urban and near-urban agriculture in a quantified case study of a globally median-sized city
Source: PLoS One. 2025 May 7;20(5):e0321203. doi: 10.1371/journal.pone.0321203 (PMC12057863; doi:10.1371/journal.pone.0321203)
Supplement: S1 Text — (DOCX) [file pone.0321203.s001.docx]

**Supporting Information**

**Resilience to Abrupt Global Catastrophic Risks Disrupting Trade: Combining Urban and Near-Urban Agriculture in a Quantified Case Study of a Globally Median-Sized City**

**Data used in the analysis of crop optimization and yield**

**Table S1: Additional data used in the analyses on potential peri-urban crops added to the list of frost-resistant crops previously analysed elsewhere [1]**

| **Crop** | **Yield used in the analysis** | **Yield data notes [all from: [2]** **unless otherwise stated]** | **Dietary energy (kJ/100g)** | **Dietary protein (kJ/100g)** | **Nutrient data notes (all data from: [3] unless indicated otherwise)** |
| --- | --- | --- | --- | --- | --- |
| Beetroot | Mid-range for roots: 60 t/ha | *“For beetroot root:* 40, 60 and 80 t/ha” (variation due to: “the target market, and whether it is a main crop or baby beetroot crop”). Our analysis was conservative in that there are also commercial yield estimates for beetroot leaves: “20, 30 and 50 t/ha.” | 132 (roots) | 1.4 (roots) | For roots we used “beetroot, peeled, fresh, raw” [X1165]. |
| Buttercup squash | Mid-point of range: 28 t/ha | For “mid-season crops with a duration of around 110 days, grown under favourable weather conditions.  These crops might achieve 24 – 32 t/ha of marketable fruit.” | 316 | 1.6 | For: “Squash, buttercup, flesh and skin, raw, fresh” [X1273] |
| Peas | 15 t/ha | “15 t/ha of peas for processing”. | 339 | 5.4 | US Department of Agriculture data for “peas, green, raw” [FDC ID: 170419]. [https://fdc.nal.usda.gov/fdc-app.html#/food-details/170419/nutrients] |
| Potatoes | Mid-point of range: 90 t/ha | “The majority of potatoes are grown through spring and summer, and their potential yields vary from about 80 t/ha for early-harvest crops through to 100 t/ha for main-crop plantings”. | 242 | 2.1 | For: “Potato, flesh & skin, raw, floury, old (October)” [X1169] |
| Sweetcorn | Mid-point of two estimates: 25 t/ha for fresh ears. This was converted to 19 t/ha of edible corn. | We took the mid-point of two NZ estimates ie, 25 t/ha:  (1) The potential yield of 20 t/ha of fresh ears. “This is typical for short-duration hybrids, and for late- or medium-duration hybrids planted in especially warm regions north of the Bay of Plenty.”  (2) Potential yield of 30 t/ha of fresh ears. “This is typical for early-planted crops of medium- to long-duration hybrids in Gisborne, Hawke’s Bay, Marlborough and Canterbury.” | 386 | 3.6 | For: “Sweet corn, kernel, fresh, raw” [X1154].  An estimated 70-80% of the weight of a fresh ear is edible corn kernels (we used 75% in our calculations). |
| Tomatoes | 103 t/ha | We averaged the “marketable yield” values as per these two scenarios:  (1) “Potential and field yield both 150 t/ha. This is typical for most of the  tomatoes grown in Hawke’s Bay provided that irrigation is scheduled and applied carefully. After allowing for fruit left in the field and factory grading, marketable  yields might be up to 120 t/ha.”  (2) “Field yield 120 t/ha due to water stress. Here water deficits are sufficient to reduce yield by 20% from a potential value of 150 t/ha. This is  representative of most tomato crops in Poverty Bay and some in Hawke’s Bay. Marketable yields might be up to 85 t/ha.” | 62 | 0.4 | For: “Tomato, red, medium to large size, fresh, ripe, raw, all year round” [X1213] |

**Table S2: Additional data on potential UA crops used in the analyses with yield data from a meta-analysis** [4]

| **Food crop** | **UA yield derived from Payen et al** **(kg/m^2^)** | **Dietary energy (kJ per 100g)** | **Dietary protein (g per 100g)** | **Comments on yield and nutrient data (all nutrient data from: [3] unless indicated otherwise)** |
| --- | --- | --- | --- | --- |
| ***Vegetables – frost resistant***[1] | | | | |
| Broccoli | 1.8 | 140 | 3.8 | Excluded from analysis as all study results involved polytunnels. Yield range was 1.0 to 3.4 kg/m^2^ for 120 study results (excluding purple sprouting broccoli and Chinese broccoli (Gai lum)). |
| Cabbages | 3.3 | 108 | 1.2 | Re-analysed yield based on 46 study results for a wide range of cabbage types (range 0.5 to 10.1 kg/m^2^). Excluded 6 results (greenhouse/hydroponic). |
| Carrots | 4.7 | 156 | 0.6 | Re-analysed yield based on 34 study results (range 0.2 to 17.6 kg/m^2^). Excluded 3 results (greenhouse/hydroponic). |
| Cauliflowers | 1.9 | 79 | 0.8 | Yield as per: “Cauliflower and broccoli” in the meta-analysis (134 study results). |
| Chicory/whitloof | 5.1 | 57 | 0.8 | Re-analysed yield based on 4 study results (range: 0.6 to 10.6) and 10 study results were excluded (hydroponics). Data was just for above ground crop (ie, ignoring the roots).  Nutrients for: “Chicory, raw” [X40]. |
| Leeks | 2.7 | 117 | 1.1 | Yield as per: "Leeks and other alliaceous vegetables" in the meta-analysis (3 study results).  Nutrients for: “Leek, stalk and bulb, fresh, raw” [X1161]. |
| Lettuce | 2.6 | 57 | 1.5 | Re-analysed yield based on 87 study results (range: 0.2 to 9.0 kg/m^2^). Excluded 137 results (aquaponics/hydroponics/greenhouse/polytunnel). |
| Onions | 1.8 | 130 | 1.4 | Yield as per: “Onions and shallots” in the meta-analysis (20 study results). |
| Spinach | 4.1 | 75 | 2.5 | Re-analysed yield based on 19 study results (range: 0.3 to 23.0 kg/m^2^). Excluded 9 hydroponic results. |
| Sugar beets | 5.3 | 180 | 1.6 | Re-analysed yield based on 8 study results (range: 5.1 to 5.5 kg/m^2^). |
| Turnips | 5.0 | 108 | 0.8 | Re-analysed yield based on 3 study results (range: 2.2 to 7.0 kg/m^2^).  Nutrients for: “raw root” [X130]. |
| ***Fruit and vegetables – frost sensitive*** | | | | |
| Aubergines/eggplants | 3.3 | 92 | 0.8 | Yield as in the meta-analysis (37 study results).  Nutrients for: “Eggplant, raw” [X1083]. |
| Beans | 1.5 | 143 | 2.1 | Re-analysed yield based on 43 study results for a wide range of bean types (range 0.0 to 9.1 kg/m^2^). The 42 results for greenhouse beans were excluded.  Nutrients for: “Bean, green runner or dwarf, seeds with pod, fresh, raw” [X1108]. |
| Courgettes | 4.6 | 56 | 1.8 | Re-analysed yield based on 23 study results (range: 0.9 to 13.4 kg/m^2^) and 2 study results were excluded (hydroponics/greenhouse).  Nutrients for: “Courgette, green, unpeeled, raw” [X1058]. |
| Cucumbers | 3.6 | 51 | 0.8 | Re-analysed yield based on 10 study results (range: 1.3 to 7.1 kg/m^2^) and 48 study results were excluded (greenhouse/artificial lighting).  Nutrients for: “Cucumber, telegraph, raw, unpeeled” [X1052]. |
| Melons | 2.3 | 134 | 1.1 | Yield as in the meta-analysis (2 study results).  Nutrients for: “Melon, rock, flesh, raw” [L106]. Using the mid-range melon type for kJ out of 3 melon types. |
| Okra | 1.1 | 111 | 2.0 | Yield as in the meta-analysis (15 study results).  Nutrients for: “Okra, raw” [X62]. |
| Peas | 3.2 | 339 | 5.4 | Re-analysed yield based on 2 study results and excluding 2 results on the more niche varieties of mange tout/sugar snap peas.  See Table A1 for nutrient data. |
| Potatoes | 4.0 | 242 | 2.1 | Re-analysed yield based on 13 study results (range: 1.1 to 8.3 kg/m^2^).  See Table A1 for nutrient data. |
| Pumpkins | 2.9 | 141 | 1.0 | Re-analysed yield based on 8 study results (range: 2.0 to 3.6 kg/m^2^).  Nutrients for: “Pumpkin, flesh, raw” [X111]. |
| Squash | 1.2 | 316 | 1.6 | Re-analysed yield based on 2 study results. See Table A1 for nutrient data. |
| Strawberries | 2.5 | 151 | 0.7 | Yield as in the meta-analysis (70 study results).  Nutrients for: “Strawberry, raw, New Zealand” [L1016]. |
| Sweet potatoes | 1.8 | 363 | 1.2 | Yield as in the meta-analysis (6 study results).  Nutrients for: “Kumara, 'Owairaka Red', flesh, raw” [X1057]. |
| Tomatoes | 8.7 | 62 | 0.4 | Yield as in the meta-analysis (208 study results).  See Table A1 for nutrient data. |
| Watermelons | 5.2 | 155 | 0.9 | Yield as in the meta-analysis (2 study results).  Nutrients for: “Watermelon, flesh, raw” [L1121]. |

**Comparison of results for UA optimized crops: comparing the meta-analysis results with a permaculture dataset**

This analysis includes the results from the meta-analysis [4] compared to yield results from a single permaculture garden in the UK [5]. The latter does not use pesticides, uses composting rather than added fertilizer, and makes some use of hoop beds and a polytunnel (eg, for tomatoes, aubergines and cucumbers; and overwintering of less hardy plants). This permaculture dataset possibly is near the peak of UA productivity as it represents work by a highly experienced and skilled professional gardener. As such it may reflect more the potential for meso-UA in converted park land organized by professional gardeners (eg, potentially employed by local government).

Comparisons of the optimal crops are shown in Table A3. The actual data used in the permaculture-related analyses and comparison with the meta-analysis yields is in Table A4.

Of note is that the permaculture yields are nearly always higher than for the meta-analysis yields (Table A3 and A4). Nevertheless, there are some similarities in optimal crop selection for both datasets (eg, peas and potatoes).

**Table S3: Results of the crop optimisation process for different settings and post-catastrophe climatic scenarios**

| **Crop category** | **Data source** | **Optimally efficient crop/s** | **Total land required** | **Extra details/comments** |
| --- | --- | --- | --- | --- |
| ***Normal climate – both frost sensitive and frost resistant crops considered*** | | | | |
| Most efficient crop/s | Meta-analysis | Peas | 292 m^2^ | At this level there was a (71%) oversupply in dietary protein (57 g per person per day). If protein requirements are ignored, peas were still the most efficient crop. In a scenario analysis, pumpkins became the most efficient crop if 75% of their foliage was spread out over paved area and only 25% was over open soil (with the latter being the standard basis for all the yield calculations in this analysis). |
| Most efficient crop/s | Permaculture | Jerusalem artichokes (sunchokes) & peas | 113 m^2^ | Jerusalem artichokes (77 m^2^) and peas (37 m^2^). If protein requirements are ignored, Jerusalem artichokes were still the most efficient crop. |
| Second most efficient crop/s | Meta-analysis | Sugar beet & spinach | 345 m^2^ | Sugar beet (326 m^2^) and spinach (18 m^2^). |
| Second most efficient crop/s | Permaculture | Potatoes & kale | 151 m^2^ | Potatoes (141 m^2^) and kale (11 m^2^). |
| Third most efficient crop/s | Meta-analysis | Potatoes | 352 m^2^ |  |
| Third most efficient crop/s | Permaculture | Salsify & parsnips | 153 m^2^ | Salsify (146 m^2^) and parsnips (7 m^2^). |
| ***Nuclear winter – only frost-resistant plants considered (no sunlight reduction adjustment)*** | | | | |
| Most efficient crop/s | Meta-analysis | Sugar beet & spinach | 345 m^2^ | Sugar beet (326 m^2^) and spinach (18 m^2^). (Same as the second choice for “normal climate”.) |
| Most efficient crop/s | Permaculture | Jerusalem artichokes & kale | 136 m^2^ | Jerusalem artichokes (94 m^2^) and kale (42 m^2^). |
| Second most efficient crop/s | Meta-analysis | Turnips & chicory/whitloof | 733 m^2^ | Turnips (417 m^2^) and chicory/whitloof (316 m^2^). |
| Second most efficient crop/s | Permaculture | Salsify & parsnip | 153 m^2^ | Salsify (146 m^2^) and parsnips (7 m^2^). |
| Third most efficient crop/s | Meta-analysis | Cabbage & carrots | 777 m^2^ | Cabbage (671 m^2^) and carrots (106 m^2^). |
| Third most efficient crop/s | Permaculture | Shallots & Brussels sprouts | 182 m^2^ | Shallots (153 m^2^) and Brussels sprouts (29 m^2^). |

**Table S4: Raw data relevant to the UA yields in the permaculture study** [5] **and comparisons with the meta-analysis yields (as per Table S2)**

| **Food crop** | **Dietary energy (kJ per 100g)** | **Dietary protein (g per 100g)** | **Permaculture yields (kg/m^2^)** | **Meta-analysis yields (kg/m^2^)** | **Extra details relating to the permaculture yield data; with nutrient data sources as per details in Table A2** |
| --- | --- | --- | --- | --- | --- |
| ***Vegetables - frost resistant*** | | | | | |
| Beetroot | 132 | 1.4 | 5.25 |  | Nutrients for: X1165. |
| Broccoli | 140 | 3.8 |  |  | Yield for: "Calabrese". |
| Brussels sprouts | 233 | 3.8 | 6 |  | Nutrients for: X1093. |
| Cabbage | 108 | 1.2 | 6 | 3.3 | Summer yield the same as for winter. |
| Carrots | 156 | 0.6 | 10 | 4.7 |  |
| Cauliflower | 79 | 0.8 | 6 | 1.9 |  |
| Celeriac | 176 | 1.5 | 7.5 |  | Nutrients for: USDA 170400. |
| Celery | 50 | 0.5 | 6 |  | Nutrients for: X1091. |
| Chard | 95 | 2.3 | 7 |  | Same nutrient value for "Swiss chard"; "Silverbeet" in NZ data.  Nutrients for: X1111. |
| Chicory/whitloof | 57 | 0.8 | 4.5 | 5.1 |  |
| Jerusalem artichokes | 304 | 2.0 | 9.6 |  | Nutrients for: USDA 169236. |
| Kale | 168 | 4.6 | 6 |  | Nutrients for: X1163. |
| Kohlrabi | 112 | 1.6 | 5.63 |  | Nutrients for: X306 (just the bulb). |
| Leeks | 117 | 1.1 | 8. | 2.7 |  |
| Lettuce | 57 | 1.5 | 7.5 | 2.6 | Yield for: "salad leaves including lettuce". |
| Onions | 130 | 1.4 | 8.75 | 1.8 |  |
| Pak choi | 72 | 2.6 | 5.25 |  | Nutrients for: X1039. |
| Parsnips | 235 | 1.0 | 10 |  | Nutrients for: X1097. |
| Radishes | 66 | 0.8 | 2 |  | Nutrients for: X1274. |
| Radishes, daikon | 76 | 0.6 | 10 |  | Nutrients for: “oriental radish”  168451 (USDA data). |
| Salsify | 343 | 3.3 | 6 |  | Nutrients for: USDA 169277. |
| Shallots | 301 | 2.5 | 6 |  | Nutrients for: USDA 170499. |
| Spinach | 75 | 2.5 | 7 | 4.1 | Yield for: "perpetual". |
| Swedes | 125 | 0.8 | 12 |  | Nutrients for: X1167. |
| Turnips | 108 | 0.8 | 7.5 | 5.0 |  |
| ***Frost-sensitive*** |  |  |  |  |  |
| Aubergines/eggplants | 92 | 0.8 | 6 | 3.3 |  |
| Beans - field | 143 | 2.1 | 4 | 1.5 |  |
| Beans - climbing | 130 | 1.8 | 5.5 |  | Nutrients for: “green string”; 1103337 (USDA). |
| Courgettes | 56 | 1.8 | 4 | 4.6 |  |
| Cucumbers | 51 | 0.8 | 24 | 3.6 |  |
| Oca (NZ yam) | 241 | 1.1 | 3.2 |  | Nutrients for: X1073. |
| Peas | 339 | 5.4 | 7.5 | 3.2 |  |
| Peppers | 85 | 0.9 | 1.5 |  | Nutrients for: “green bell”; X1049. |
| Potatoes | 242 | 2.1 | 9 | 4.0 | Yield for: "main crop". |
| Pumpkins | 141 | 1.0 | 5 | 2.9 |  |
| Squash | 316 | 1.6 | 5 | 1.2 | Yield for: "winter". |
| Tomatoes | 62 | 0.4 | 12 | 8.7 |  |

**References**

1. Wilson N, Payne B, Boyd M. Mathematical optimization of frost resistant crop production to ensure food supply during a nuclear winter catastrophe. Scientific Reports. 2023;13(1):8254.

2. Reid JB, Morton JD. Nutrient management for vegetable crops in New Zealand: Horticulture New Zealand Wellington; 2019.

3. The New Zealand Institute for Plant and Food Research Limited and the Ministry of Health (New Zealand). The New Zealand Food Composition Database. The New Zealand Institute for Plant and Food Research Limited and the Ministry of Health (New Zealand), (2022). <https://www.foodcomposition.co.nz/>.

4. Payen FT, Evans DL, Falagán N, Hardman CA, Kourmpetli S, Liu L, et al. How much food can we grow in urban areas? Food production and crop yields of urban agriculture: a meta‐analysis. Earth's future. 2022;10(8):e2022EF002748.

5. Richards H, Cooper S. The Self-Sufficiency Garden: Dorling Kindersley Ltd; 2024.
