# Supplementary material for: Resilience to abrupt global catastrophic risks disrupting trade: Combining urban and near-urban agriculture in a quantified case study of a globally median-sized city
Source: PLoS One. 2025 May 7;20(5):e0321203. doi: 10.1371/journal.pone.0321203 (PMC12057863; doi:10.1371/journal.pone.0321203)
Supplement: Attachment — Submitted filename: Review Comments PONE-D-24-50856).docx [file pone.0321203.s002.docx]

**Review Comments: Manuscript** #: PONE-D-24-50856

**MS Title**: Resilience to Abrupt Global Catastrophic Risks Disrupting Trade: Combining Urban and Near-Urban Agriculture in a Quantified Case Study of a Globally Median-Sized City

**General comments**

The manuscript addresses an increasingly critical issue of food security in the face of abrupt global catastrophic risks (GCRs), a topic of both scientific and societal importance. The study's focus on a globally median-sized city and its integration of multiple factors, including urban, meso-urban, and near-urban agriculture, is innovative and adds value to the existing literature. The use of Google Earth image analysis to quantify available urban land and the optimization of crops for specific scenarios demonstrate a strong methodological framework. It provides actionable insights, such as the area requirements for urban and near-urban agriculture, and recommendations for municipal land-use policies to enhance resilience to GCRs. The study contains experiments conducted appropriately, and the methodologies have been well elaborated. The results of the study have also been well explained and discussed properly. However, there are many weaknesses in the write-up which need to be improved before publication.

**Specific comments**

Shortcomings in each section are mentioned separately here under:

**Title**: Appropriate.

**Abstract**

While detailed, the abstract may overwhelm readers with too much technical information. Consider simplifying it to emphasize the key findings and their significance more succinctly.

**Keywords:** Appropriate.

**Introduction**

The introduction is comprehensive but could benefit from better structuring to ensure clarity. The paragraphs introduce many related but distinct ideas, which can be overwhelming. Consider breaking the text into smaller, clearly delineated sections.

Terms like "micro-UA," "meso-UA," and "near-urban agriculture" are introduced but used interchangeably with other terms like "peri-urban agriculture." Ensure consistent terminology to avoid confusion.

The transition from the introduction to the methods section could be improved. Consider ending the introduction with a clear summary of the study's aims, framing how the methods will address the identified gaps.

**Methods**

**Study Site Description**

Consider briefly explaining why this specific population size or climate is particularly relevant to the study's goals. Clarify if the selection of the city impacts the generalizability of the findings to other regions with different population sizes or climates.

**Optimization of Crops for Urban Agriculture (UA)**

The assumption of one crop cycle per year might be limiting, acknowledge this and consider discussing potential implications in scenarios where multiple crop cycles are feasible. Provide a rationale for excluding certain crops (e.g., herbs, garlic, cereals) from the micro-/meso-UA analysis. This will help readers understand any potential biases or limitations.

**Land Availability Analysis**

Including examples or supplementary data on inter-rater reliability metrics would strengthen this section. Consider adding more details about the extrapolation method used for scaling sampled residential lot data to the city level. For example, were any socioeconomic or geographic factors considered during extrapolation?

**Food Production Estimation**

It would be helpful to mention any uncertainties or assumptions in the yield data (e.g., variations in soil quality or local climate conditions). Clarify how the nuclear winter scenarios (e.g., 150 Tg vs. 5 Tg soot scenarios) were integrated into the yield calculations. Did they involve specific yield reduction coefficients?

**Near-Urban Cropping Needs**

The inclusion of biofuel feedstock connection to the core research questions should be more explicit. Briefly justify why biofuel considerations are essential in the context of urban agriculture. Highlight any assumptions or trade-offs made in estimating the diesel and land requirements for near-urban cropping and biofuel production.

**Statistical Analysis:** Appropriate

**Results**

Ensure these figures are visually intuitive, with clear labels and legends to aid interpretation of the data, particularly for readers less familiar with the topic. The discussion of fuel requirements and biofuel feedstock is relevant but could benefit from further elaboration on the environmental implications or potential trade-offs.

References to supporting information (e.g., Table S3) are helpful, but the main text should briefly explain why this additional data is significant. Ensure that the results are tied back to the study's overarching goals and research questions. Highlight how these findings advance the understanding of urban agriculture and food security.

**Discussion**

The text is dense and requires clearer subheadings for accessibility. Divide the discussion into more distinct sections with descriptive subheadings, such as "Urban Agriculture Potential," "Challenges and Limitations," "Policy Recommendations," and "Future Research Directions." This will improve readability and highlight key arguments.

The text provides numerical estimates without sufficient contextualization. Add comparative statements or visual aids (e.g., tables, graphs) to contextualize figures like the 1249 ha of near-urban land or 134,000 L of biodiesel. For example, compare these numbers with the total available land in a typical city or provide percentages relative to current agricultural outputs.

Policy suggestions lack actionable specifics. Provide concrete examples of successful urban or near-urban agricultural policies from similar contexts. For instance, reference case studies where zoning laws or urban farming incentives have been effectively implemented.

The discussion is focused on a specific case study without broader generalizations. Discuss how findings could apply globally, particularly to cities in varying climates or socioeconomic settings. Highlight adaptations required for regions with extreme conditions or limited resources

**Conclusion:** Appropriately written.

**References:** Check the reference style as per the journal format.
