# Supplementary material for: Resilience to abrupt global catastrophic risks disrupting trade: Combining urban and near-urban agriculture in a quantified case study of a globally median-sized city
Source: PLoS One. 2025 May 7;20(5):e0321203. doi: 10.1371/journal.pone.0321203 (PMC12057863; doi:10.1371/journal.pone.0321203)
Supplement: Attachment — Submitted filename: REVIEWR COMMENTS PLOS ONE UA.docx [file pone.0321203.s003.docx]

**Topic**

**Resilience to Abrupt Global Catastrophic Risks Disrupting Trade:
Combining Urban and Near-Urban Agriculture in a Quantified Case Study
Of a Globally Median-Sized City.**

The topic is too wordy, consider revising it to make it simpler.

**Abstract**

1. The study calculated land space for urban agriculture (UA) using Google Earth Image analysis however, the author failed to state the method used to determine the optimum food crops by dietary calorie energy (kJ) and protein level.
2. Revise and indicate all the methods.

**I. Introduction**

1. The author's justification for the paper is not enough, particularly the weak link of UA to global catastrophe risk (GCR).
2. Again, the author failed to pose relevant research questions to direct the focus of the study. Rather, the questions are under the methods section (see 2.2, 2.3…).
3. Research questions must come before the objective of the study
4. I suggest you revise the introduction

**Methods**

**2.2 What are the optimal crops to grow in the UA and near-urban spaces?**

1. What is the theory behind using linear programming with an Excel solver in a study such as this? The author must explain the rationale for using the method relative to other methods in the literature.
2. In lines 189 - 191, the author “considered crop yield (kg/tonne of harvested crop per land area) and provision of dietary energy (kJ) and dietary protein at levels to feed an average New Zealander” Where did the author pick the data from? Who cultivates the crops, the farmer or the author? The explanation is not clear.
3. How was the data obtained for the nutrient level analysis? Was it a laboratory analysis? Explain.
4. Check line 198. “where at least two study results were reported in the meta-analysis and…..”The sentence is incomplete and not clear

**2.3 How much land is available for UA in this median-sized case-study city?**

1. Using orthorectified Google Earth Images to determine the land available for UA	is satisfactory.

**Table 2:** Key variables considered in the analysis

The author needs to consider re-constructing Table 2 for clarity.

2.0 **Results**
2.1 **Optimized cropping**

1. In the methodology, the author's objective was to estimate the optimum yield of dietary energy and protein, but the results focused on only the optimum crop yield (lines 318 – 324). Why did that happen? How were the nutrient levels for the crops analysed?
2. Why peas and not sugar beet and spinach per Figure 2? Is it based on the nutrient level (dietary energy and protein)? Explain
3. Again, the results have not been compared to the relevant existing literature. Provide the literature to back your findings.
   2.2 **Potential micro- and meso-UA land area**
1. Again these results are not compared to the existing relevant literature.
2. The author should consider splitting Table 3 into 4 tables each with a heading and explanation for clarity.
   4.0 **Discussion**
1. The author failed to situate the discussion based on the reviewed literature. Provide relevant references
   4.1 **UA inputs**
1. The discussions from lines 494 – 592 on input requirements for UA were neither shown in the literature nor the findings but are discussed. What is the justification for these discussions?
2. The author may need to revisit the entire work, from the literature review to the methods and findings, to reflect what is discussed.

**5.0 Conclusion**

1. The author’s conclusion is merely a recap of the key findings and not a conclusion.
2. This section should capture both conclusions and recommendations.
